# A nomogram for predicting paradoxical immune reconstitution inflammatory syndrome associated with cryptococcal meningitis among HIV-infected individuals in China

**DOI:** 10.1186/s12981-022-00444-5

**Published:** 2022-04-26

**Authors:** Xiaoxu Han, Hui Liu, Yuqi Wang, Peng Wang, Xin Wang, Yunyun Yi, Xin Li

**Affiliations:** 1 Department of Integrated Traditional Chinese and Western Medicine, Beijing Ditan Hospital, Capital Medical University, 8 Jingshundong Street, Chaoyang District Beijing, 100015 People’s Republic of China; 2 Department of Integrated Traditional Chinese and Western Medicine, Peking University Ditan Teaching Hospital, Beijing, 100015 People’s Republic of China

**Keywords:** HIV, Immune reconstitution inflammatory syndrome, Cryptococcal meningitis, Risk factors, Nomogram, Prediction model, Risk stratification

## Abstract

**Background:**

Cryptococcal meningitis (CM) associated immune reconstitution inflammatory syndrome (CM-IRIS) is the second most common complication in HIV-infected individuals with cryptococcal meningitis, with a reported mortality rate ranging from 8 to 30%. Given the devastating consequences of CM-IRIS related intracranial neuroinflammation and its challenging in diagnosis, we conducted a study to explore the risk factors and the occurrence of paradoxical CM-IRIS in HIV-infected patients, which is of great value for prevention and clinical management.

**Methods:**

We conducted a retrospective cohort study to identify the indicators associated with paradoxical CM-IRIS among 86 HIV-infected patients with CM using univariate and multivariate cox analysis. A nomogram was constructed using selected variables to evaluate the occurrence of paradoxical CM-IRIS at 6 months and 12 months after ART initiation. The discrimination and calibration of the nomogram were assessed by concordance index (C-index) and calibration plots. Decision curves analysis (DCA) were used to evaluate clinical effectiveness of the nomogram. Subsequently, to help clinicians recognize patients at high risk faster, patients were divided into high-risk and low-risk groups according to the best cutoff point identified by X-tile.

**Results:**

Of 86 AIDS patients with CM, 22.1% experienced paradoxical CM-IRIS at a median of 32 days after antiretroviral therapy (ART) initiation. The occurrence of paradoxical CM-IRIS was associated with age, ART initiation within 4 weeks of antifungal treatment, a four-fold increase in CD4 T cell counts, C-reactive protein levels, and hemoglobin levels independently. These five variables were further used to construct a predictive nomogram. The C-index (0.876) showed the favorable discriminative ability of the nomogram. The calibration plot revealed a high consistency between the predicted and actual observations. DCA showed that the nomogram was clinically useful. Risk stratification based on the total score of the nomogram showed well-differentiated in the high-risk and low-risk groups. Clinicians should pay attention to patients with total points high than 273.

**Conclusions:**

We identified the predictive factors of paradoxical CM-IRIS and constructed a nomogram to evaluate the occurrence of paradoxical CM-IRIS in 6 months and 12 months. The nomogram represents satisfactory performance and might be applied clinically to the screening and management of high-risk patients.

## Introduction

Cryptococcal meningitis (CM) is a severe opportunistic infection in the central nervous system. It is regarded as the most common cause of meningitis in adults living with HIV [1]. It mainly manifests with mental status changes, headache, fever, and even consciousness disorders or seizures, which are life-threatening if anti-CM treatment is delayed. Vision and hearing loss also occurrs in these patients due to increased intracranial pressure and fungal burden [2, 3]. Rapid cerebrospinal fluid (CSF) cryptococcal antigen detection is recommended as the preferred diagnostic method when people with HIV are suspected of having CM. The serum cryptococcal antigen assay is an alternative preferred diagnostic approach when the lumbar puncture is limited or clinically contraindicated [4]. A short course (2 weeks) of induction therapy with amphotericin B plus flucytosine followed by prolonged fluconazole therapy is the most agreed upon treatment guideline recommendation [5, 6].

For HIV-infected individuals, initiation of antiretroviral therapy (ART) within 7 days of HIV diagnosis is recommended by the WHO, effectively suppressing viral replication and leading to CD4 cell recovery. However, HIV-infected patients should first undergo clinical examination (for symptoms and signs of tuberculosis or CM) to evaluate for significant opportunistic infections before ART initiation. Immediate ART initiation is not recommended among HIV-infected patients with CM because of the risk of increased mortality, which may be caused by paradoxical immune reconstitution inflammatory syndrome [4]. After ART initiation, some HIV-infected individuals with CM present with an excessive inflammatory response and experience clinical deterioration, despite microbiological improvement of effective antifungal treatment. This condition was defined as paradoxical CM-associated immune reconstitution inflammatory syndrome (CM-IRIS) [7, 8].

CM-IRIS is reported in 30% of patients with CM and usually occurs within the first month of ART initiation. Patients who developed paradoxical CM-IRIS experienced ART-associated immune imbalance with failed re-establishment of the Th1 and Th2/17 balance [9], resulting in clinical deterioration and increased mortality. A delay in ART (4–6 weeks following ART initiation) is recommended to decrease the incidence of paradoxical CM-IRIS. However, treatment access and timing are challenging in places where medical resources are scarce, leading to increased incidence and mortality of paradoxical CM-IRIS. Existing evidence reveals paradoxical CM-IRIS, a life-threatening complication, with a reported mortality rate of 8–30% [1, 9]. The absence of laboratory tests in clinical practice to confirm paradoxical CM-IRIS makes diagnosis and management difficult. Thus, identifying predictive factors of paradoxical CM-IRIS is critical for prevention, diagnosis, and management.

Many studies have identified the risk factors for CM-IRIS, including severe immunosuppression with CD4 cell counts lower than 100 cells per μL, the timing of ART initiation, C-reactive protein (CRP) level, and high cryptococcal fungal burden at ART initiation [10–12]. However, the risk factors for developing paradoxical CM-IRIS in China remain obscure and require further study, to improve clinical decision-making. Nomograms are helpful and readily available models that can be scored by integrating significant risk factors, which is convenient for clinicians to predict prognosis and individual treatment plans in numerous diseases [13, 14]. Some studies have used nomograms to predict the incidence and mortality of meningitis [15, 16]. However, to our knowledge, no studies use nomograms to predict the occurrence of paradoxical CM-IRIS in HIV-infected patients. This study aims to identify the risk factors for paradoxical CM-IRIS and establish an effective predictive model for evaluating the incidence of paradoxical CM-IRIS in HIV-infected patients.

## Methods

### Subjects

We performed a retrospective study in an observational cohort. This study was approved by the institutional review board (IRB) of Beijing Ditan Hospital, affiliated with Capital Medical University. We enrolled patients diagnosed with acquired immune deficiency syndrome (AIDS) with CM infection between January 2009 and December 2019 at Beijing Ditan Hospital. The inclusion criteria were as follows: (1) AIDS diagnosis and management according to *The Guidelines on HIV/AIDS Diagnosis and Treatment in China (2018 edition*) [17] recommended by the China Center of Disease Control. CSF ink staining positive or cryptococcal antigen-positive, (2) without ART before hospitalization, and (3) patients with good adherence. The exclusion criteria were as follows: (1) pregnancy, breastfeeding, and severe comorbidities (such as sepsis and acute respiratory distress); (2) patients with malignant tumors; and (3) incomplete medical history.

### Treatment criteria

Amphotericin B plus flucytosine or fluconazole plus flucytosine were used as induction regimens for treating CM among patients with AIDS. Fluconazole was used for maintenance [17]. All patients with CM had an initial lumbar puncture while receiving antifungal treatment, regardless of symptoms or signs of intracranial hypertension. ART based on two nucleoside reverse transcriptase inhibitors (NRTIs) was initiated after the patient's condition was stable. For patients with other opportunistic infections (such as tuberculosis and cytomegalovirus), standardized treatment was performed according to *the Guidelines on HIV/AIDS Diagnosis and Treatment in China (2018 edition*) [17].

### Data collection

We collected baseline data, including primary demographic data (age, sex, complications, and other opportunistic infections), clinical symptoms, signs, and intervention records. Laboratory data included blood biochemical tests (such as red blood cell (RBC) counts and CRP), immunological examination (such as initial CD4 cell counts, HIV-RNA load), pathogenetic detection (CSF cryptococcal antigen and CSF India ink test), and CSF biochemical detection. Imaging data, including cranial nuclear magnetic resonance imaging or computed tomography. In addition, the time when paradoxical CM-IRIS occurred was recorded. For patients with paradoxical CM-IRIS, CD4 cell counts were recorded at baseline and the onset of paradoxical CM-IRIS. Data were obtained at baseline and 3 months after ART initiation for patients without paradoxical CM-IRIS.

### Definition

The diagnosis of paradoxical CM-IRIS was adjudicated by two experienced clinicians, and was based on the deterioration of clinical manifestations (such as fever, eye disease, and recurrence of symptoms following ART without microbiological evidence), changes in CSF open pressure, increase in CD4 cell counts and decrease in HIV RNA load [4, 18]. The diagnosis of consciousness disorders was adjudicated by experienced clinicians based on whether the subject had clinical manifestations such as lethargy, coma, confusion and delirium. The outcome event of Cox proportional hazard analysis was defined as paradoxical CM-IRIS occurring 12 months after ART initiation.

### Statistical analysis

SPSS (version 25.0) software (IBMCorp., Armonk, N.Y., USA) was used for data analysis. Statistics were performed between two groups: patients with and without paradoxical CM-IRIS. For continuous variables, normally distributed data are described as the means ± standard deviation, and group comparisons were performed by t test. Nonnormally distributed data were described by the median and interquartile range (IQR), and the nonparametric rank-sum test was used to compare groups. In addition, percentages were used to describe categorical variables, and the χ^2^ test was used to compare groups. The results were considered statistically significant at p < 0.05.

R version 4.1.0 (R Core Team (2021), Vienna, Austria) was used to carry out univariate and multivariate Cox analysis and nomogram construction. The multivariate Cox model was built using the variables with p < 0.1 in univariate cox regression to identify the independent predictive factors of paradoxical CM-IRIS. The selected variables were included in the nomogram. The incidence of paradoxical CM-IRIS within 6 months and 12 months of ART initiation was predicted using the nomogram. The Bootstrap repeated sampling method was used in performing internal validation by repeating sampling 1000 times. Concordance index (C-index) and calibration plot were used to evaluate the discrimination and calibration of the nomogram. Decision curve analysis (DCA) can assess whether risk models help us make a better clinical decision [19] by quantifying net benefits at ranges of reasonable risk thresholds. The Kaplan–Meier (K-M) method evaluated risk stratifications with the nomogram to further verify the model's effectiveness and help clinicians identify high-risk individuals faster. X-tile was used to screen out the best cutoff point of risk stratification [20].

## Results

### The clinical feature of AIDS patients with CM and developed with paradoxical CM-IRIS or not in the baseline

A total of 86 AIDS patients with CM were enrolled in our cohort study, including 77 males and 9 females, and the median age of patients was 35 years. The patients had varying degrees of headache (88.4%), nausea (62.8%), vomiting (59.3%), consciousness disorder (25.6%), convulsions (22.1%), visual impairment (23.3%), hearing impairment (7.0%), and other symptoms. All patients had not received ART at the time of admission, and antifungal treatment was given after the diagnosis of CM.

In our study, 19 patients were diagnosed with paradoxical CM-IRIS (22.1%) at a median of 32 days (IQR, 19–55 days) after ART initiation, including 1 female and 18 males. Sixty-seven patients did not meet the diagnostic criteria for paradoxical CM-IRIS, including 59 males and 8 females. The age of patients with paradoxical CM-IRIS (median age of 28 years, IQR, 25–35 years) was younger than that of patients without paradoxical CM-IRIS (median age of 35 years, IQR, 28–43 years) (p = 0.028). The incidence of an altered level of consciousness in patients with paradoxical CM-IRIS was significantly lower than those without CM-IRIS (p = 0.044). There was no significant difference in CSF protein, CSF glucose, and CSF pressure between the two groups. At the same time, CSF chloride was lower in patients with paradoxical CM-IRIS (p = 0.019) than in the other group. Compared to those without CM-IRIS, patients with paradoxical CM-IRIS had significantly lower hemoglobin (HGB) levels and RBC counts (p = 0.005 and p = 0.047, respectively), while CRP was increased considerably (p = 0.012). There was no significant difference in blood biochemistry, initial CD4 cell counts, and HIV viral load between the two groups. Additionally, 68.4% of patients with paradoxical CM-IRIS after initiating ART within 4 weeks of antifungal treatment, while only 19.4% in patients without paradoxical CM-IRIS did (p < 0.001). The differences in baseline clinical characteristics between the two groups are shown in Table 1.

**Table 1:** The baseline characteristics of patients with paradoxical CM-IRIS or not

Characteristics	Non-CM-IRIS patients (n = 67)	CM-IRIS patients (n = 19)	Statistic	p-value
Gender, n (%)				
Male	59 (88.1)	18 (94.7)	0.704	0.401
Female	8 (11.9)	1 (5.3)		
Age (years)	35 (28–43)	28 (25–5)	− 2.194	**0.028**
Complication, n (%)				
Hepatitis	9 (13.4)	2 (10.5)	0.112	0.738
Syphilis	8 (11.9)	5 (26.3)	2.384	0.123
Tuberculosis	7 (10.4)	4 (21.1)	1.492	0.222
Mycotic infection	5 (26.3)	25 (37.3)	0.788	0.375
Diabetes	1 (1.5)	1 (5.3)	0.926	0.336
Hypertension	2 (3.0)	1 (5.3)	0.228	0.663
Symptoms, n (%)				
Headache	60 (89.6)	17 (89.5)	0.000	0.992
Nausea	39 (58.2)	15 (78.9)	2.725	0.099
Vomit	36 (53.7)	14 (73.7)	2.421	0.120
Visual impairment	16 (23.9)	4 (21.1)	0.066	0.797
Hearing impairment	4 (6.0)	2 (10.5)	0.473	0.491
Consciousness disorder	23 (34.3)	2 (10.5)	4.067	**0.044**
Head imaging abnormal, n (%)	13 (31.0)	6 (13.6)	3.743	0.053
CSF tests				
CSF WB counts (cells/μL), n (%)				
< 10	10 (14.9)	2 (10.5)	4.006	0.261
10–49	29 (43.3)	12 (63.2)		
50–200	19 (28.4)	5 (26.3)		
> 200	9 (13.4)	0 (0.0)		
CSF protein (mg/dL)	42.1 (29.4, 72.2)	33.7 (25.5, 53.9)	− 0.921	0.357
CSF glucose (mmol/L)	2.63 ± 0.11	2.82 (1.91, 3.26)	0.321	0.755
CSF chloride (mmol/l)	120 ± 0.72	115.7 (113.2–120)	− 2.353	**0.019**
CSF pressure, (mmH2O), n (%)				
≤ 180	17 (25.4)	2 (10.5)	1.909	0.385
181–250	14 (20.9)	5 (26.3)		
> 250	36 (53.7)	12 (63.2)		
Blood biochemical tests				
RBC (10^12^/L), n (%)				
< 4.0	22 (32.8)	11 (57.9)	3.931	**0.047**
≥ 4.0	45 (67.2)	8 (42.1)		
HGB (g/L)	124.3 ± 2.6	106.2 ± 7.8	3.642	**0.005**
WBC (10^9^/L)	5.14 (3.75, 6.17)	5.33 (3.62, 7.43)	− 0.645	0.519
Neutrophils (10^9^/L)	4.02 (2.73, 6.31)	3.62 (1.96, 4.74)	− 1.343	0.179
Lymphocyte (10^9^/L)	0.62 (0.47, 0.90)	0.68 ± 0.08	− 0.359	0.719
Monocyte (10^9^/L)	0.35 (0.24, 0.50)	0.42 ± 0.06	0.370	0.712
ALT (U/L)	23.7 (14.7, 38.2)	18.4 (14.9, 25.5)	− 1.239	0.215
AST (U/L)	21.7 (14.9, 36.8)	21 (17.3, 28.1)	− 0.005	0.996
Albumin (g/L)	36.19 ± 0.63	34.88 ± 1.13	0.993	0.323
Globulin (g/L)	36.11 ± 0.86	38.45 ± 1.27	− 1.332	0.186
A/G	1.05 ± 0.04	0.92 ± 0.05	1.794	0.076
CRP (mg/L)	9.50 (3.00–30.80)	24.10 (11.40–70.70)	2.514	**0.012**
ESR (mm/h)	45.06 ± 3.50	60.47 ± 8.18	− 1.957	0.054
Immunological detection				
HIV viral load (copies/mL)	175,690 (73,200, 377,237)	87,657 (53,735, 1,076,291)	− 0.161	0.872
Initial CD4 cells counts (cells/ul)	19 (8–34)	15 (5–25)	− 1.021	0.307
CD4 cells counts after ART (cells/ul)	59 (22–109)	45 (16–86)	− 0.973	0.330
Increase in CD4 cell counts, n (%)				
≤ 4 folds	44 (65.7)	7 (36.8)	5.098	**0.024**
> 4 folds	23 (34.3)	12 (63.2)		
Therapeutic schedule, n (%)				
AmB ± 5FC	3 (4.5)	2 (10.5)	3.481	0.481
FLU ± 5FC	29 (43.3)	7 (36.8)		
Voriconazole	6 (9.0)	1 (5.3)		
Mixed regimens	23 (34.3)	5 (26.3)		
Non-standard treatment	6 (9.0)	4 (21.1)		
Initiate HAART time, n (%)				
≤ 4 weeks	13 (19.4)	13 (68.4)	16.862	** < 0.001**
> 4 weeks	54 (80.6)	6 (31.6)		
ART regimens, n (%)				
2NRTIs + 1INRTIs	60 (89.6)	17 (89.5)	0.323	0.851
2NRTIs + 1PIs	6 (9.0)	2 (10.5)		
2NRTIs + 1TNSTIs	1 (1.4)	NA		

The significance of bold emphasis indicate that this variable has a statistical difference between the two groups

### Risk factors for paradoxical CM-IRIS selected by univariate and multivariate cox analysis

Variables with significant differences at baseline were included in the univariate and multivariate cox analyses. Using multivariate cox analysis, we identified that initiating ART within 4 weeks of antifungal treatment (Hazard Ratio (HR) 7.073, 95% CI 2.472–20.242, p < 0.001), and a four-fold increase in CD4 cell counts after initiation of ART (HR 3.055, 95% 1.092–8.546, p = 0.033) and CRP (HR 1.010, 95% 1.004–1.021, p = 0.002) were risk factors for the development of paradoxical CM-IRIS. Initiating ART within 4 weeks of antifungal treatment increased the risk of paradoxical CM-IRIS by 7.07 times. Patients with a higher than four-fold increase in CD4 cell counts after ART had 3.06 times higher odds of developing CM-IRIS. For every 1 mg/L increase in CRP, the risk of paradoxical CM-IRIS increased by 1.01 times. Contrastingly, age and HGB were protective factors for developing of paradoxical CM-IRIS. For every 1 year increase of age and every 1 g/L increase in HGB, the risk of CM-IRIS reduced by 0.91 and 0.98 times, respectively (Table 2).

**Table 2:** Univariate and multivariate Cox analysis of variables associated with the presence of paradoxical CM-IRIS

Variables	Univariate analysis		p-value	Multivariate analysis		p-value
	HR	95% CI		HR	95% CI	
Age	0.946	(0.894–1.000)	0.051	0.910	(0.846–0.980)	**0.013**
Consciousness disorder						
Yes	1.077	(0.390–3.000)	0.887			
No	1.000					
Head imaging abnormal						
Yes	0.436	(0.166–1.147)	0.093	0.547	(0.193–1.551)	0.257
No	1.000			1.000		
Initiate HAART time						
≤ 4 weeks	6.036	(2.289–15.920)	< 0.001	7.073	(2.472–20.242)	** < 0.001**
> 4 weeks	1.000			1.000		
Increase in CD4 cell counts						
≤ 4 folds	1.000	(0.944–6.095)	0.066	1.000	(1.092–8.546)	**0.033**
> 4 folds	2.399			3.055		
HGB	0.978	(0.959–0.997)	0.030	0.980	(0.962–0.998)	**0.031**
CRP	1.010	(1.003–1.014)	0.004	1.013	(1.004–1.021)	**0.002**

The significance of bold emphasis indicate that this variable has a statistical difference between the two groups

### Nomogram construction and validation

**Fig. 1:**
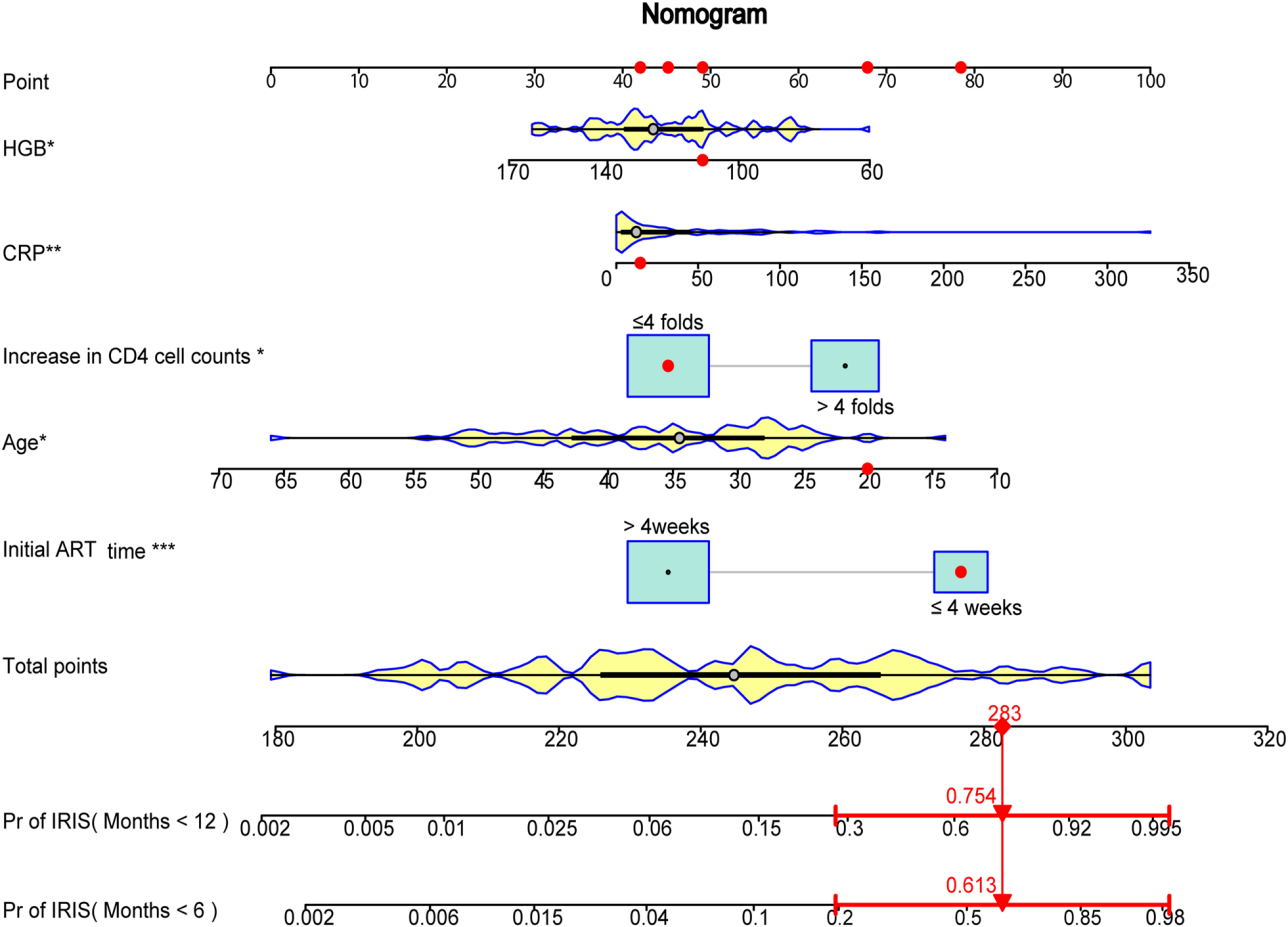
An example used the nomogram to predict the occurrence of paradoxical IRIS in 6 months and 12 months. The predictive nomogram consists of HGB, CRP, increase in CD4 cell counts, age, and initial ART time. This patient was 20 years old, CRP of 14.5 mg/L, HGB of 111 g/L, who initiated ART within 4 weeks after antifungal treatment and increased in CD4 cell counts lower than four-folds. According to the nomogram, the total score of this patient added up to 283, suggesting the probability of paradoxical CM-IRIS in 6 months and 12 months was 0.613 and 0.754, respectively

Based on the results of multivariate cox analysis, 5 variables, including age, time of ART initiation, increase in CD4 cell counts, CRP, and HGB were incorporated to construct a predictive nomogram for predicting the probability of developing CM-IRIS within 6 months and 12 months of ART initiation. Figure 1 shows how to use the nomogram to predict the probability of developing CM-IRIS in a given patient (Fig. 1). The total score was the cumulative sum of the individual scores from 5 variables. Patients in our study had total risk scores ranging from 180 to 320.

**Fig. 2:**
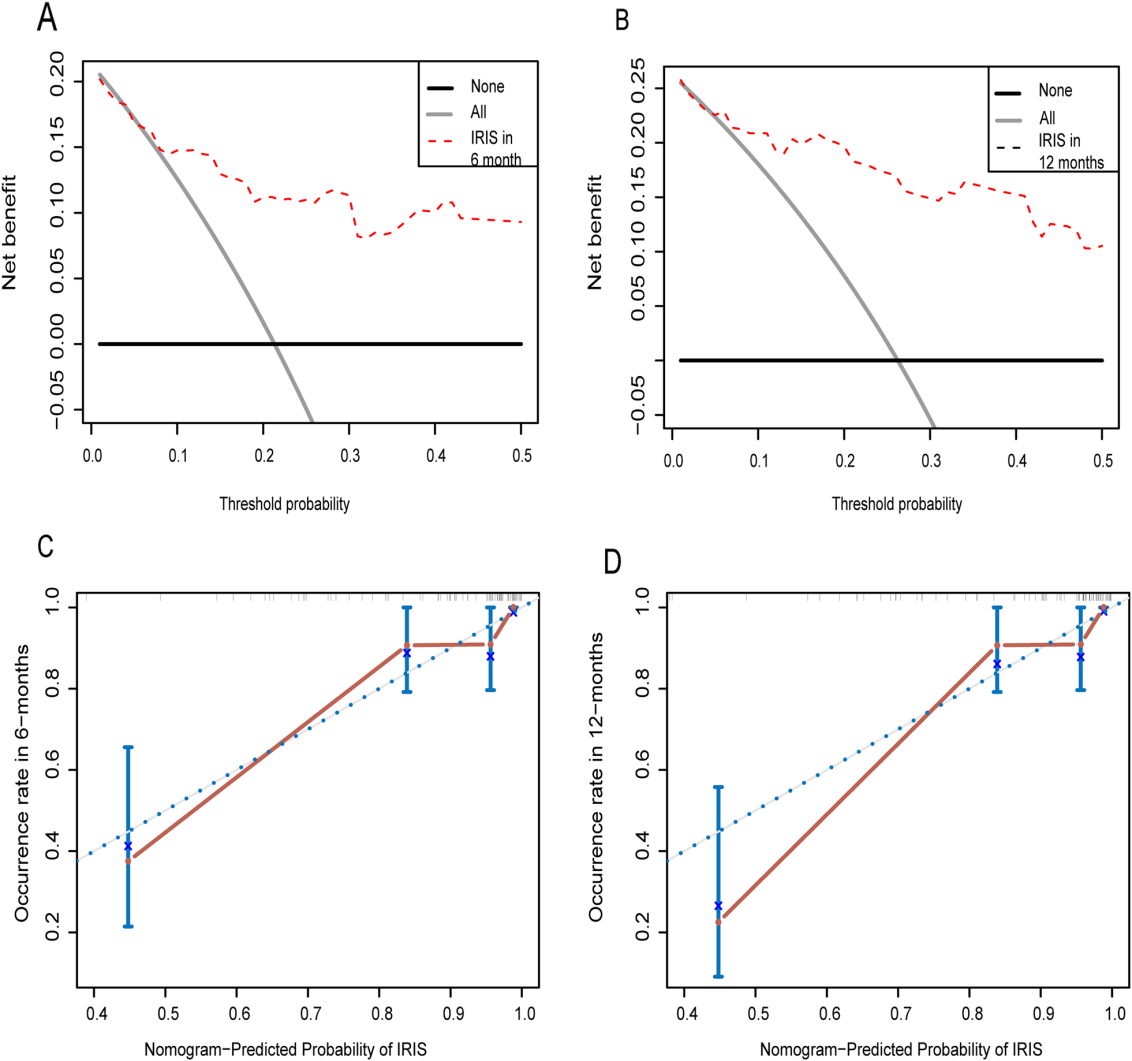
Validation of the nomogram. **A** Decision curves analysis of the nomogram to predict the occurrence of paradoxical CM-IRIS in 6 months. **B** Decision curves analysis of the nomogram to predict the occurrence of paradoxical CM-IRIS in 12 months. The calibration curves for 6 months (**C**) and 12 months (**D**) probability of paradoxical CM-IRIS. The blue lines represent the ideal reference line, and the red lines represent actual observation

The performance of the nomogram was assessed by C-index, DCA curve, and calibration plots. The C-index value was 0.876, and the DCA curve revealed that the net benefit of the nomogram was high within the threshold range of 0.1 to 0.5 (Fig. 2A, B), indicating high discrimination and clinical practicality of the nomogram. Calibration plots showed high consistencies between the predicted probability of developing CM-IRIS and actual observation (Fig. 2C, D). In summary, the nomogram had an excellent performance.

### Risk stratification based on the nomogram

**Fig.3:**
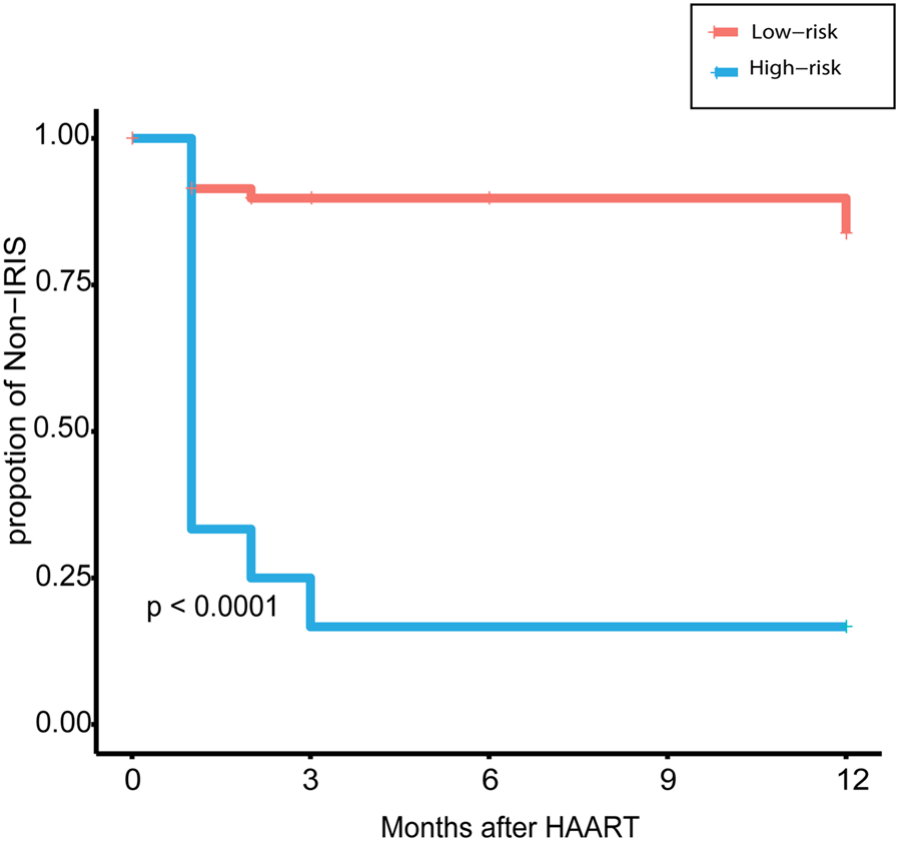
Kaplan–Meier curve of patients with different risk-stratified. Patients were divided into high-risk and low-risk groups according to the total point of the nomogram. Kaplan–Meier curve estimates the incidence of paradoxical CM-IRIS between two groups within 12 months from ART initiation to paradoxical CM-IRIS. The blue line represents the group with a high risk of paradoxical CM-IRIS, and the red line represents the group at low risk. A p-value was lower than 0.05 means that the two groups were well-differentiated

Risk stratification was based on the overall score of the nomogram. Patients were divided into two groups according to the best cutoff value selected by X-tile: low risk (total points < 273) and high risk (total points ≥ 273). K-M curve showed that the two groups of patients were well-differentiated (Fig. 3). Risk stratification helps clinicians identify high-risk patients more quickly and perform individualized treatment.

## Discussion

In this retrospective study, we identified several clinical predictors associated with the occurrence of paradoxical CM-IRIS in HIV-infected patients using Cox regression analysis. Consequently, age, time of ART initiation, increased CD4 cell counts, HGB, and CRP were identified and used to construct a predictive nomogram. The validation study of this nomogram demonstrated its discriminative and calibration capabilities.

Our study found that age was the independent risk factor for paradoxical CM-IRIS. Some studies found that the population of naïve T cells reduced with advancing age, and younger HIV-infected patients on ART had faster CD4 cell recovery [21, 22]. In addition, a study revealed that younger HIV-infected patients with CM responded better to antifungal therapy [23]. Therefore, younger HIV-infected patients with CM may have a more robust and faster immune restoration after ART initiation, which could play an essential role in the overexpressed inflammatory response and paradoxical clinical deterioration. Our results reveal that younger patients have a higher risk of paradoxical CM-IRIS, consistent with the conclusions mentioned above.

A systematic review showed increased mortality among people living with HIV who initiated ART within 4 weeks of antifungal treatment, which may relate to a potentially high risk of CM-IRIS [24]. In addition, a retrospective cohort study in China confirmed that ART initiation within 4 weeks of antifungal treatment was associated with paradoxical CM-IRIS also [11]. Consistently, we found that ART initiation within 4 weeks of antifungal treatment increased the risk of paradoxical CM-IRIS by 7.07 times, which may further reinforce the previous conclusions. Therefore, ART initiation time should be deferred to 4–6 weeks after antifungal treatment to reduce the poor prognosis caused by CM-IRIS.

Our report identified that a four-fold increase in CD4 cell counts was a risk factor for paradoxical CM-IRIS. Similar to our results, a previous study revealed that IRIS was associated with an increase in CD4 cell percentage and CD4 to CD8 ratio after 1 month of ART [25]. An increase in CD4 cell counts reflects rapid immune recovery among HIV-infected patients, associated with higher CSF fungal clearance and better protection against opportunistic infection [26]. However, increasing evidence reported that CD4 cells might contribute to clinical deterioration in HIV-infected patients with CM. Therefore, monitoring CD4 cells counts was needed to reduce the risk of paradoxical CM-IRIS. We did not find an association between initial CD4 cell counts and paradoxical CM-IRIS. This may be because all enrolled patients were hospitalized, and most of them were admitted with CD4 cells less than 50 cells/μL.

Low HGB reflected the presence of disseminated opportunistic infection, chronic inflammation, or advanced HIV. A previous study had identified the effect of low HGB in tuberculosis-IRIS, mycobacterial-IRIS, and CM-IRIS [27–29]. These associations were consistent with our results. HGB detection is simple and widely used so that it may become a critical serological marker for paradoxical CM-IRIS.

CRP is an acute-phase inflammatory protein that is a significant surrogate marker for inflammation or infection. It is mainly generated occurs in hepatocytes in response to the induction of interleukin-6 [30, 31]. A prospective cohort study has revealed the critical role of interleukin-6 and CRP in IRIS events. Patients who developed IRIS had higher levels of CRP before ART initiation, and interleukin-6 levels progressively increased on ART until the development of IRIS, compared with controls [32]. Other studies have reported similar increases in CRP and interleukin-6 in some pathogen-specific IRIS parthenogenesis [33, 34]. Our results revealed that CRP was a risk factor for paradoxical CM-IRIS, consistent with the conclusions above. Thus, dynamic monitoring of CRP may be a credible and straightforward approach to prevent and manage paradoxical CM-IRIS events.

Because of the devastating consequence of IRIS-related intracranial neuroinflammation, it is crucial to diagnose and manage it early. We propose a nomogram composed of easily obtained indicators to evaluate the relative risk of paradoxical CM-IRIS. This is the first nomogram to predict the occurrence of paradoxical CM-IRIS in Chinese HIV-infected patients. It had a good performance in predicting the 6 months and 12 months probability of incidence of CM-IRIS. Patients were divided into two groups: high-risk and low-risk groups according to the nomogram, which was of great value for clinical reference and decision processes. It might help clinicians identify early patients at high risk of paradoxical CM-IRIS and intensify clinical follow-up or individualized treatment as a guide [35].

The present study had some limitations. First, the size of this study was relatively small and from a single institution, which may have some inevitable bias. Therefore, future research should validate our findings using a more extensive database from multiple institutions. Second, due to the limitations of retrospective studies, the occurrence of paradoxical CM-IRIS may be underestimated. Despite these limitations, we explored the risk factors of paradoxical CM-IRIS in HIV-infected patients and constructed a predictive model with good performance. We hope that this model might help clinical practice manage HIV-infected patients with CM and improve the prognosis of patients.

## Conclusion

In conclusion, our study identified five variables associated with paradoxical CM-IRIS using univariate and multivariate cox analysis. Initiating ART within 4 weeks of antifungal treatment, a four-fold increase in CD4 cell counts and high CRP increased the risk of paradoxical CM-IRIS among HIV-infected patients. Contrarily, HGB and age were protective factors for paradoxical CM-IRIS. Notably, we constructed a nomogram to evaluate the occurrence of paradoxical CM-IRIS in 6 months and 12 months and divided patients into high-risk and low-risk groups according to the nomogram. It helps clinicians to identify high-risk patients and develop personalized treatment.

## Data Availability

The datasets generated during and/or analyzed during the current study are available from the corresponding author on reasonable request.

## Abbreviations

CM: Cryptococcal meningitis
CM-IRIS: Cryptococcal meningitis associated immune reconstitution inflammatory syndrome
C-index: Concordance index
DCA: Decision curves analysis
ART: Antiretroviral therapy
CSF: Cerebrospinal fluid
CRP: C-reactive protein
IRB: Institutional review board
AIDS: Acquired immune deficiency syndrome
NRTIs: Nucleoside reverse transcriptase inhibitors
RBC: Red blood cell
IQR: Interquartile range
K-M: Kaplan–meier
HGB: Hemoglobin
HR: Hazard ratio
